# Detecting gene-gene interactions for complex quantitative traits using generalized fuzzy classification

**DOI:** 10.1186/s12859-018-2361-5

**Published:** 2018-09-18

**Authors:** Xiangdong Zhou, Keith C. C. Chan

**Affiliations:** 1 College of Mathematics and Computer Science, Fuzhou University, Fuzhou, Fujian China; 2 Department of Computing, the Hong Kong Polytechnic University, Kowloon, Hong Kong China

**Keywords:** Quantitative traits, Gene-gene interactions, Multifactor dimensionality reduction, Ordinal traits, Fuzzy accuracy

## Abstract

**Background:**

Quantitative traits or continuous outcomes related to complex diseases can provide more information and therefore more accurate analysis for identifying gene-gene and gene- environment interactions associated with complex diseases. *Multifactor Dimensionality Reduction* (MDR) is originally proposed to identify gene-gene and gene- environment interactions associated with binary status of complex diseases. Some efforts have been made to extend it to *quantitative traits* (QTs) and ordinal traits. However these and other methods are still not computationally efficient or effective.

**Results:**

*Generalized Fuzzy Quantitative trait MDR* (GFQMDR) is proposed in this paper to strengthen identification of gene-gene interactions associated with a quantitative trait by first transforming it to an ordinal trait and then selecting best sets of genetic markers, mainly *single nucleotide polymorphisms* (SNPs) or *simple sequence length polymorphic markers* (SSLPs), as having strong association with the trait through generalized fuzzy classification using extended member functions. Experimental results on simulated datasets and real datasets show that our algorithm has better success rate, classification accuracy and consistency in identifying gene-gene interactions associated with QTs.

**Conclusion:**

The proposed algorithm provides a more effective way to identify gene-gene interactions associated with quantitative traits.

## Background

With the advent of the genomic era, doctors can utilize genetic data to analyze the mechanisms of diseases and customize medical treatment. Diseases are usually associated with genetic variants, mainly *single nucleotide polymorphisms* (SNPs) or *simple sequence length polymorphic markers* (SSLPs), which are already a valuable source for mapping complex diseases and complex genetic traits [1]. Searching for genetic factors that influence complex traits and complex diseases is both a goal and a challenge for modern geneticists.

In recent years, the field has been revolutionized by using *genome-wide association studies* (GWASs) to assess the statistical associations of genetic variants with many important common diseases [2]. A single-locus approach, where each variant is tested individually for association with a specific phenotype is used by most of these studies. However research limited to individual gene effects will make a large proportion of the heredity of complex diseases and complex traits unexplained [3, 4]. Gene-gene and gene-environment interactions play an important role in genetic association studies of complex diseases and complex traits [5]. If a genetic factor functions primarily through interaction with other genetic factors or environmental factors, the effect might be missed if the gene is examined individually without allowing for its interactions with these other unknown factors.

A variety of methods have been proposed to identify gene-gene interactions existing in complex diseases. These methods include regression modeling [6–10], data reduction [11–14], genetic programming [15], neural networks [16, 17], pattern mining [18, 19] and machine learning approaches, such as random forest [20], support vector machine [21] and ensemble learning [22].

These methods are mainly used in a case control study to identify interactive SNPs for predicting a binary disease status and have achieved great success. Among these methods, the *Multifactor Dimensionality Reduction* (MDR) method, was proposed as a nonparametric and model-free data reduction approach for identifying interactions without significant main effects and has been successfully applied to identify gene-gene interactions in many common complex diseases [13, 23, 24]. In the analysis of binary traits, MDR reduces high dimension of multi-locus genotype combinations to one dimension of two groups: high risk group and low risk group, thus avoids the problem of sparse data combinations and models with too many parameters. Each genotype combination is classified as either high risk or low risk according to its ratio of cases and controls. The set of genetic markers which has best classification performance is then selected as having the strongest association with the trait. Although MDR has been extended in many directions, it is mainly applied in binary traits.

However in many cases, continuous outcomes or quantitative traits such as body weight, tumor size, blood pressure can provide more accurate analysis.

Some efforts have been made to extend MDR to *quantitative traits* (QTs). The *Combinatorial Partitioning Method* (CPM) [25] was proposed to identify partitions of multi-locus genotypes for predicting variation in quantitative trait levels. The *Restricted Partition Method* (RPM) detects multi-locus genotypes as predictors of a quantitative trait by a partitioning of genotypes into subgroups. The *Generalized MDR* (GMDR) [26] extends MDR to continuous phenotypes and includes covariate adjustment. In *Model based MDR* (MB-MDR) [27], MDR is extended to continuous outcomes by using parametric regression.

There are also methods based on information theory. In [28], a method built on two information-theoretic metrics, the *k*-way interaction information (KWII) and phenotype-associated information (PAI) is developed for analyzing the gene-gene and gene-environmental interactions associated with quantitative traits. In [29], as an extension of the usefulness of information gain, a nonparametric evaluation method of conditional entropy of a quantitative phenotype associated with a given genotype is proposed. In [30], an entropy-based statistic which asymptotically follows a χ^2^ distribution is proposed to test genetic epistasis. This approach can test genetic epistasis with high efficiency in a case-only design.

CPM searches over the state space made up of all possible sets of genotypic partitions of all the *m*-locus genotypes to identify *m* loci that divide corresponding genotypes into *k* partitions that are most similar within and most dissimilar between partitions for the mean of a quantitative trait. The number of *k* sets of genotypic partitions is a Stirling number of the second kind:

1 $$ S\left({g}_{M,}k\right)=\frac{1}{k!}\sum \limits_{i=0}^{k-1}{\left(-1\right)}^i\left(\begin{array}{c}k\\ {}i\end{array}\right){\left(k-i\right)}^{g_M} $$

Where *g*_*M*_ is the size of the set of *m*-locus genotypes. A permutation test is used to estimate *P* values for the *R*^2^ for each of the *k* sets of genotypic partitions.

The RPM tries to find the most reasonable partition for evaluation to decrease most of the computational burden associated with the CPM. However a permutation test is used for all possible *m*-locus classifiers.

MB-MDR is implemented in R (https://www.r-project.org/) but is only used on one-way and two-way interaction models [30]. G-MDR still requires the outcome in the data file to be dichotomous [30].

KWII needs to compute the entropies of all subsets of *m* loci. Although the computation of the PAI requires only individual and joint entropies, making it computationally far more tractable than the KWII, the hill climbing algorithm it employs will miss many interactions which have small main effects.

In *Quantitative MDR* (QMDR) [31], to exploit continuous outcomes to make the analysis more accurate, a test statistic, rather than the balanced accuracy, is used to determine the best interaction model. This is a computationally efficient algorithm. However this method still classified the outcome into two groups: high and low level groups, which results in the loss of the large variability of the quantitative outcome.

Also there are few methods applied to ordinal categorical traits. Ordinal categorical traits such as the obesity classification based on body mass index (e.g., normal, pre-obese, mild obese and severe obese), the diabetes diagnosis based on glucose level (e.g., normal, impaired glucose tolerance and diabetes) are common in many genetic association studies. These traits are also derived from quantitative traits. In *Ordinal MDR* (OMDR) [32], MDR is extended to analyze gene-gene interaction for ordinal traits and tau-b [33], a common ordinal association measure, is used to replace balanced accuracy to evaluate interactions. However the tau-b measure only measures the degree of tendency of positive association between true categories of an ordinal trait and predicted categories and doesn’t consider the difference between true categories and predicted categories.

In order to better use the information contained in the quantitative trait, we first classify the quantitative outcome into several (greater than two) ordinal levels. Then an extended MDR is used to identify gene-gene interactions on this converted ordinal categorical trait. Rather than using balanced accuracy or common ordinal association measures, such as tau-b, we use a generalized fuzzy classification method to select the set of genetic markers as having the strongest association with the trait. Usually for each prediction of a category, its accuracy value is either 1, if the prediction is right, or 0, if the prediction is wrong. However for quantitative or ordinal traits, when the prediction is wrong, the closeness of a quantitative value to the true category is different. To reflect such difference, member functions of fuzzy sets could be employed to compute accuracy in classification. Since the range of a member function is between 0 and 1, to better describe the difference of quantitative values to a category, we extend its range to {− 1, 1} when it is used in fuzzy classification.

In this paper, a new kind of member functions which have an extended output range from − 1 to 1 are proposed to be used in fuzzy classification first. Then *Generalized Fuzzy Quantitative MDR* (GFQMDR) algorithm which is an improvement of Fuzzy Quantitative trait based Ordinal MDR (FQOMDR) in [34] is given to strengthen identification of gene-gene interactions associated with QTs. This algorithm first transforms a quantitative trait into an ordinal trait and then select best sets of SNPs as having strong association with the trait using such kind of member functions in the extended MDR. To test the performance of the proposed algorithm, we use it to identify five different interaction models in simulated data and compare success rates with three other methods. We also use it in two real data sets to select SNPs having strong association with the trait and compare balanced test accuracy and consistency with the same three other methods.

## Methods

### Traditional member functions

The degree of membership of different values to a fuzzy set can be computed using a membership function whose range is between 0 and 1.

**Fig. 1:**
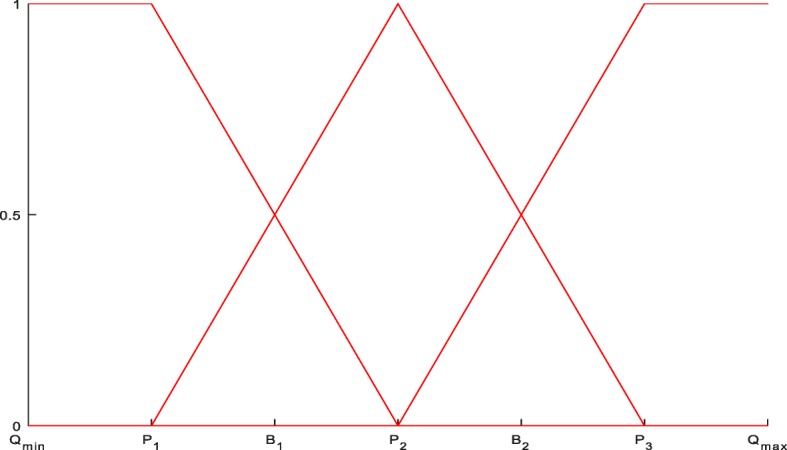
The linear membership functions of high(H), average(A) and low(L) levels of a QT

Take QTs as an example. Usually we can divide them into three intervals or levels: high (H), average (A) and low (L) associated with three fuzzy sets. Here as an example, we use equal length intervals and associate them with three fuzzy sets using linear member functions, as shown in Fig. 1.

Let *Q*_min_ and *Q*_max_ denote the maximum and minimum values that a QT takes on in all samples in a dataset. *B*_1_ and *B*_2_ are upper borders of the low level and the average level respectively. *P*_1_, *P*_2_ and *P*_3_ are the middle positions of the low level, average level and high level respectively can be derived as follows:

2 $$ {P}_1=\frac{Q_{\mathrm{min}}+{B}_1}{2} $$

3 $$ {P}_2=\frac{B_1+{B}_2}{2} $$

4 $$ {P}_3=\frac{B_2+{Q}_{\mathrm{max}}}{2}. $$

Then member functions for *L*, *A* and *H* levels in Fig. 1 can be expressed as:

5 $$ {\mu}_{L1}(x)=\left\{\begin{array}{l}1,\kern4.5em \mathrm{if}\kern0.5em x<={P}_1\\ {}\frac{P_2-x}{P_2-{P}_1},\kern1.5em \mathrm{if}\kern0.5em {P}_1<x<={P}_2\\ {}0,\kern4em \mathrm{otherwise}\end{array}\right. $$

6 $$ {\mu}_{A1}(x)=\left\{\begin{array}{l}\frac{x-{P}_1}{P_2-{P}_1},\kern1.5em \mathrm{if}\kern0.5em {P}_1<=x<={P}_2\\ {}\frac{P_3-x}{P_3-{P}_2},\kern1.5em \mathrm{if}\kern0.5em {P}_2<x<={P}_3\\ {}0,\kern4em \mathrm{otherwise}\end{array}\right. $$

7 $$ {\mu}_{H1}(x)=\left\{\begin{array}{l}0,\kern4em \mathrm{if}\kern0.5em x<={P}_2\\ {}\frac{x-{P}_2}{P_3-{P}_2},\kern1.5em \mathrm{if}\kern0.5em {P}_2<x<={P}_3\\ {}1,\kern4em \mathrm{otherwise}\end{array}\right. $$

### Generalized fuzzy classification using extended member functions

Membership functions of fuzzy sets can also be used as an accuracy measure in fuzzy classification. For example, when different values are classified to the high level, we can get different accuracies between 0 and 1 from *μ*_*H1*_(*x*). However when selecting a best classifier composed of a set of SNPs to classify a QT, such a range could not fully show differences among different classifiers. For example, if there are both 500 samples in genotypes that are classified as the high level for two classifiers, for classifier 1 there are 300 samples located at P_3_, 200 samples located at P_2_ and 100 samples located at P_1_ in genotypes that are classified as the high level, for classifier 2 there are 300 sample located at P_3_, 100 samples located at P_2_ and 200 samples located at P_1_ in genotypes that are classified as high levels, then the accuracies of the high level for these two classifiers would be the same: 0.6. However classifier 1 is obviously a better classifier to classify the high level. To reflect such difference, we extend the range of member functions from {0, 1} to {− 1, 1} when they are used in fuzzy classification to select the best classifier.

**Fig. 2:**
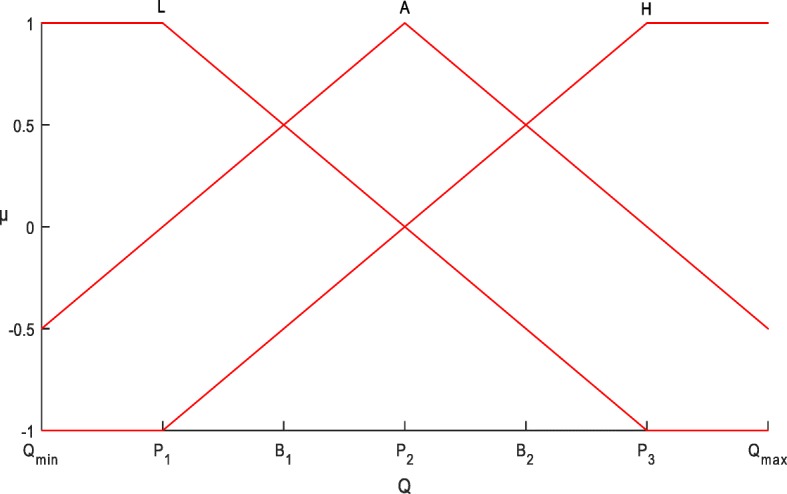
The extended linear membership functions of high(H), average(A) and low(L) levels of a QT

Such a linear extended member function is illustrated in Fig. 2 and can be expressed as:

8 $$ {\mu}_{L2}(x)=\left\{\begin{array}{l}1,\kern4.5em \mathrm{if}\kern0.5em x<={P}_1\\ {}\frac{P_2-x}{P_2-{P}_1},\kern1.5em \mathrm{if}\kern0.5em {P}_1<x<={P}_3\\ {}\hbox{-} 1,\kern3.5em \mathrm{otherwise}\end{array}\right. $$

9 $$ {\mu}_{A2}(x)=\left\{\begin{array}{l}\frac{x-{P}_1}{P_2-{P}_1},\kern2em \mathrm{if}\kern0.5em x<={P}_2\\ {}\frac{P_3-x}{P_3-{P}_2},\kern2em \mathrm{otherwise}\end{array}\right. $$

10 $$ {\mu}_{H2}(x)=\left\{\begin{array}{l}\hbox{-} 1,\kern3.5em \mathrm{if}\kern0.5em x<={P}_1\\ {}\frac{x-{P}_2}{P_3-{P}_2},\kern1.5em \mathrm{if}\kern0.5em {P}_1<x<={P}_3\\ {}1,\kern4.5em \mathrm{otherwise}\end{array}\right. $$

It can also be regarded as a transformation of the member function in Fig. 1 as follows:

11 $$ {\mu}_{L2}(x)=\left\{\begin{array}{l}{\mu}_{L1(x)},\kern2.5em \mathrm{if}\kern0.5em x<={P}_2\\ {}\frac{P_2-x}{P_2-{P}_1},\kern1.5em \mathrm{if}\kern0.5em {P}_2<x<={P}_3\\ {}\hbox{-} 1,\kern3.5em \mathrm{otherwise}\end{array}\right. $$

12 $$ {\mu}_{A2}(x)=\left\{\begin{array}{l}\frac{x-{P}_1}{P_2-{P}_1},\kern1.5em \mathrm{if}\kern0.5em x<={P}_1\kern0.5em \\ {}{\mu}_{A1(x)},\kern2.5em \mathrm{if}\kern0.5em {P}_1<=x<={P}_3\\ {}\frac{P_3-x}{P_3-{P}_2},\kern1.5em \mathrm{otherwise}\end{array}\right. $$

13 $$ {\mu}_{H2}(x)=\left\{\begin{array}{l}\hbox{-} 1,\kern3.5em \mathrm{if}\kern0.5em x<={P}_1\\ {}\frac{x-{P}_2}{P_3-{P}_2},\kern1.5em \mathrm{if}\kern0.5em {P}_1<x<={P}_2\\ {}{\mu}_{H1(x)},\kern2.5em \mathrm{otherwise}\end{array}\right. $$

### MDR algorithm

In order to detect high- dimensional gene-gene interaction, MDR reduces genotype combinations at multiple loci into a single class variable taking values of either high risk or low risk categories, then tests association between a binary trait or disease with this new one dimensional variable.

The MDR method proceeded as follows. The 10-fold cross validation is used. A set of *m* genetic factors is selected and their possible combinations or cells are represented in *m* dimensional space. For example, for two diallelic loci, each has three genotypes and there are nine two-locus-genotype combinations. Then the ratio of the number of cases to the number of controls is estimated within each cell, which is then labeled either as “high-risk”, if the cases:controls ratio is equal or greater than some threshold, or otherwise as “low-risk”. Thus all cells are allocated to either high risk group or low risk group, which reduces the *n*-dimensional model into a one dimensional model. The procedure is repeated for each possible *n*-factor combination. The training balanced accuracy of the two groups is used to select the best classifier. Balanced accuracy is defined as the arithmetic mean of sensitivity and specificity:

14 $$ \left(\mathrm{sensitivity}+\mathrm{specif}\ \mathrm{icity}\right)/2=\left(\mathrm{TP}/\left(\mathrm{TP}+\mathrm{FN}\right)+\mathrm{TN}/\left(\mathrm{TN}+\mathrm{FP}\right)\right)/2 $$

where TP represents true positives, TN represents true negatives, FP represents false positives, and FN represents false negatives. The prediction error of the selected best classifier can be estimated using the remaining one-tenth of the data to get the testing balanced accuracy. The process is repeated for all ten training sets and testing sets and for each of the selected *m*-locus classifiers, the number of cross-validation replicates in which it is chosen as the best classifier (cross-validation consistency) is recorded. The *m*-locus classifier that has the maximum testing balanced accuracy and highest cross-validation consistency is selected as the final best *m*-locus classifier, where cross-validation consistency is used as a tie-break.

For an ordinal categorical trait with *J* levels, an *m* dimensional cell is labeled as one of *J* groups as follows. Let 1, 2, ..., *J* be *J* levels or categories for an ordinal trait. For any combination of *m* SNPs, let *n*_*+j*_ be the number of individuals in class *j*, *n*_*ij*_ be the number of individuals with the *i*th multi-locus genotype in category *j*, where *i* = {1, 2,...,3^*m*^} and j = 1, 2,..., *J*. Then the *i*th m-locus genotype will be labeled as category *c*(*i*) as follows:

$$ c(i)=\underset{j\in \left\{1,\dots, J\right\}}{\arg \max}\left(\frac{n_{ij}}{n_{+j}}\right) $$

### GFQMDR algorithm

GFQMDR extends MDR to analyze quantitative traits by first converting them to ordinal traits. Then Instead of evaluating each classifier using balanced accuracy or common ordinal association measures, it uses generalized fuzzy classification based on extended member functions to evaluate each classifier and select the best one as having the strongest association with the trait. The procedure of GFQMDR is as follows:

1. Divide the range of a quantitative trait into *J* intervals and label them as categories 1, 2,…,*J* respectively.
2. Partition the dataset into *L* subsets for *L*-fold *cross-validation* (CV). Use one of the *L* subsets as a testing set and the rest as a training set.
3. For each *m*-way interaction derived from *m* SNPs or SSLPs, let *n*_*ij*_ be the number of individuals belonging to category *j* with the *i*th multi-locus genotype in the training set, *n*_*+j*_ be the total number of individuals belonging to category *j* in the training set, where *i* = {1, 2,...,3^*m*^} and *j* = 1, 2,..., *J*. Then all individuals with the *i*th multi-locus genotype will be assigned into the category *c*(*i*) by the classifier corresponding to the *m* given SNPs as follows:
  15 $$ c(i)=\underset{j\in \left\{1,\dots, J\right\}}{\arg \max}\left(\frac{n_{ij}}{n_{+j}}\right) $$

where *n*_*ij*_ and *n*_*+j*_ are real numbers, *n*_*ij*_ is computed using the extended linear member function, *n*_*+j*_, the size of class *j*, is computed using the traditional linear member function.

4. Compute the training balanced accuracy for each *m*-way interaction:
  16 $$ \frac{1}{J}\sum \limits_{i=1}^{3^m}\frac{n_{i,c(i)}}{n_{+c(i)}} $$

where *n*_*i,c*(*i*)_, the number of individuals with the *i*th multi-locus genotype which really belong to the class they are classified to, is computed using the extended linear member function.

5. Select *k* classifiers that have best training balanced accuracies and compute their testing balanced accuracies.
6. Repeat steps 3–5 on all *L* CV dataset.
7. Since multiple gene-gene interactions associated with a QT is common in complex traits, multiple candidates of *m*-way gene-gene interactions are selected as having the maximum testing balanced accuracy and highest generalized cross-validation consistency based on top-*K* selection (GCVC^*K*^ or simplified as GCVC) [34], where general cross-validation consistency is used as a tie-break.. The GCVC^*K*^ is calculated as follows:
  17 $$ {\mathrm{GCVC}}^K={\sum}_{l=1}^L{I}_l\ \mathrm{where}\ {I}_l=\left\{\begin{array}{l}1,\kern1.25em \mathrm{if}\ \mathrm{the}\ \mathrm{MDR}\ \mathrm{classifier}\ \mathrm{is}\ \mathrm{identified}\ \mathrm{as}\ \mathrm{one}\ \\ {}\kern1.5em \mathrm{of}\ \mathrm{top}\hbox{-} K\ \mathrm{classifier}\mathrm{s}\ \mathrm{at}\ {1}^{\mathrm{th}}\mathrm{CV}\ \mathrm{dataset}\\ {}0,\kern1.25em \mathrm{otherwise}\end{array}\right. $$
8. To lower type I error, compute *P* values of selected candidates of *m*-way gene-gene interactions based on 1000 permutations and select candidates having *P* values smaller than α (α is a prescribed threshold) as final identified gene-gene interactions.

## Results

### Experiments on simulation data

#### Experimental setup

The simulation experiment is designed to study the success rate of the proposed method and compare it with that of MDR, OMDR and *Fuzzy Quantitative MDR* (FQMDR) which uses fuzzy classification based on traditional member functions.

**Fig. 3:**
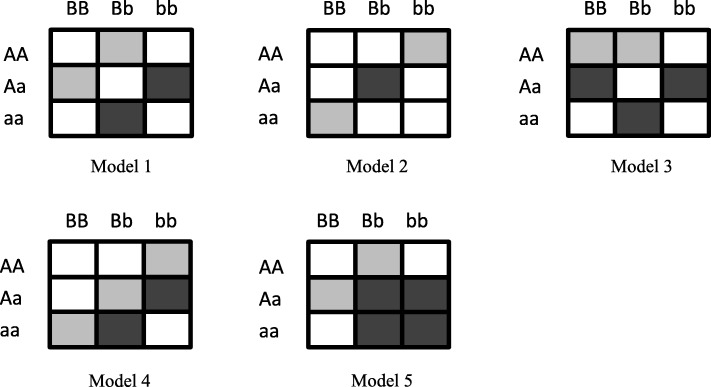
Models of two way interactions for ordinal traits. White, light grey, dark grey represent normal, low risk, high risk of an ordinal trait respectively. (Figure is from [25])

Five different interaction models were used for the ordinal trait transferred from a quantitative trait (Fig. 3) [32]. For each model, one pair of SNPs was simulated as a causal factor among all possible combinations.

The program gs 2.0 [35, 36] can quickly generate a large number of samples with genotype data based on real data that share similar local *linkage disequilibrium* (LD) patterns as those in human populations. It can be used to implement various interaction models. So we first use gs 2.0 to generate simulated genotype data.

Since the outcome is binary status (case or control), we derive continuous outcome from the penetrance functions (the penetrance function denotes the probability of being a case for each genotype combination.) of the five models as follows:

Let *f*_*ij*_ be the element from the *i*th row and *j*th column of a penetrance function for two interacting SNPs, the QT is generated from the following normal distribution:

18 $$ \mathrm{y}\mid \mathrm{SNP}1=i,\mathrm{SNP}2=j\sim \mathrm{N}\left({f}_{ij},{\sigma}^{\ast}\right) $$

where *f*_*ij*_ and σ* are the mean and variance of the normal distribution respectively. Then the QT is transferred to an ordinary trait with three categories. Since the QT obeys a normal distribution, we use the following classification. Let *μ*, *σ* be the mean value and variance of the quantitative trait, any quantitative trait value smaller than *μ*-*σ*/2 is classified as low category; any value between *μ*-*σ*/2 and *μ* + *σ*/2 is classified as middle category; any value larger than *μ* + *σ*/2 is classified as high category.

We use two different *minor allele frequencies* (MAF = 0.2 and 0.4), five different variances (σ* = 0.1, 0.2, 0.3, 0.4 and 0.5) and three different sample size (*n* = 200, 400, 800) with fixed SNP number (100 SNPs) and penetrance functions (0.01, 0.25, 0.5 for white, light grey, dark grey in Fig. 3. respectively) to create simulated datasets. For each interaction model, 100 replicated datasets were generated. Varying variances with fixed penetrance functions is equivalent to varying penetrance functions with fixed variances.

Hit ratio which is defined as the proportion of replicates with which the true causal SNPs are detected as the best SNPs among all possible same number of SNPs is used to measure the success rate. Here the best SNPs are also selected by using step 8 of the GFQMDR algorithm with α set as 0.01.

To test the type I error rate, the null datasets with no causal pair of SNPs were simulated for different sample sizes (*n* = 200, 400 and 600) and different SNP numbers (*m* = 10, 15, 20). Permutation *P* values of the identified strongest interaction pair of SNPs were calculated by permuting trait values of each dataset 1000 times. The ratio of the permutation 푃 values smaller than the significance level 훼=0.01 in 1000 replicates is calculated as the type I error rate. The number of the permutation ensured its accuracy to one decimal place when expressed in percent.

To demonstrate the power of the proposed algorithm to detect multiple gene-gene interactions associated with a QT, we use a combination of two models to simulate two set of two interacting SNPs associated with a QT. We use two combinations. One is a combination of model 1 and model 2, another is a combination of model 3 and model 4.

Let *f*_1*ij*_ be the element from the *i*th row and *j*th column of the penetrance functions for the first set of two interacting SNPs and *f*_2*kl*_ be the element from the *k*th row and *l*th column of the penetrance functions for the first set of two interacting SNPs, the QT is generated from the following normal distribution:

$$ \mathrm{y}\mid \mathrm{SNP}1=i,\mathrm{SNP}2=j,\mathrm{SNP}1=k,\mathrm{SNP}2=l\sim \mathrm{N}\left({w}_1\ {f}_{1 ij}+{w}_2\ {f}_{2 ij},{\sigma}^{\ast}\right) $$

where *w*_1_*f*_1*ij*_ + *w*_2_*f*_2*ij*_ and σ* are the mean and variance of the normal distribution respectively, *w*_1_ and *w*_2_ are the weights. Then the QT is transferred to an ordinary trait with three categories as in the first experiment.

We use two different MAFs (0.2 and 0.4), three different variances (σ* = 0.1, 0.2 and 0.3) and three ratio of weights with fixed sample size (*n* = 200) and fixed SNP number (100 SNPs) and penetrance functions (0.01, 0.25, 0.5 for white, light grey, dark grey in Fig. 3. respectively) to create simulated datasets. For each interaction model, 100 replicated datasets were generated. Hit ratio is also used to measure the success rate with α set as 0.01 in step 8 of the GFQMDR algorithm here.

#### Experiment results

Experiment results of five models are shown in Tables 1, 2, 3, 4 and 5.

**Table 1:** Hit ratios (%) for model 1

Sample size	MAF	Method	Variance				
			0.1	0.2	0.3	0.4	0.5
200	0.2	GFQMDR	82	57	25	11	6
		FQMDR	81	51	23	9	5
		OMDR	64	55	29	16	6
		MDR	78	47	18	7	3
	0.4	GFQMDR	99	79	56	36	25
		FQMDR	99	66	45	26	17
		OMDR	97	72	47	27	17
		MDR	94	67	43	18	14
400	0.2	GFQMDR	98	76	48	31	11
		FQMDR	98	76	53	28	16
		OMDR	90	73	46	28	19
		MDR	96	68	45	23	11
	0.4	GFQMDR	100	89	74	54	43
		FQMDR	99	83	65	44	31
		OMDR	100	81	61	43	37
		MDR	99	75	57	41	24
800	0.2	GFQMDR	100	90	71	53	33
		FQMDR	100	92	67	49	36
		OMDR	89	86	63	50	35
		MDR	99	87	60	48	31
	0.4	GFQMDR	100	99	96	89	80
		FQMDR	100	95	91	73	62
		OMDR	100	98	83	71	60
		MDR	100	95	82	66	55

**Table 2:** Hit ratios (%) for model 2

Sample size	MAF	Method	Variance				
			0.1	0.2	0.3	0.4	0.5
200	0.2	GFQMDR	90	66	44	22	10
		FQMDR	89	58	38	24	13
		OMDR	89	62	35	23	11
		MDR	82	59	29	15	10
	0.4	GFQMDR	97	82	61	42	28
		FQMDR	96	77	54	41	30
		OMDR	93	80	55	41	30
		MDR	90	69	52	38	19
400	0.2	GFQMDR	98	84	71	52	34
		FQMDR	97	82	66	52	36
		OMDR	99	78	63	48	35
		MDR	92	80	56	43	31
	0.4	GFQMDR	99	95	81	66	49
		FQMDR	98	92	78	63	51
		OMDR	98	92	78	72	52
		MDR	97	91	73	64	48
800	0.2	GFQMDR	100	96	89	74	49
		FQMDR	100	96	88	70	56
		OMDR	100	95	85	68	53
		MDR	99	94	84	63	51
	0.4	GFQMDR	100	100	94	82	68
		FQMDR	100	100	93	83	74
		OMDR	100	100	90	83	74
		MDR	100	98	91	76	67

**Table 3:** Hit ratios (%) for model 3

Sample size	MAF	Method	Variance				
			0.1	0.2	0.3	0.4	0.5
200	0.2	GFQMDR	93	65	44	21	9
		FQMDR	90	52	28	13	7
		OMDR	87	50	22	11	6
		MDR	87	52	24	10	4
	0.4	GFQMDR	83	73	55	37	27
		FQMDR	83	70	53	37	24
		OMDR	80	65	52	40	31
		MDR	80	60	44	30	13
400	0.2	GFQMDR	99	79	61	41	27
		FQMDR	95	66	45	26	11
		OMDR	98	64	34	18	15
		MDR	96	61	32	14	5
	0.4	GFQMDR	100	92	83	70	56
		FQMDR	99	91	75	58	48
		OMDR	100	91	74	55	46
		MDR	96	89	72	54	36
800	0.2	GFQMDR	100	99	85	64	44
		FQMDR	100	95	76	53	40
		OMDR	100	89	72	37	34
		MDR	99	91	71	44	23
	0.4	GFQMDR	1	1	97	93	81
		FQMDR	1	1	93	86	73
		OMDR	1	99	93	82	72
		MDR	1	1	94	82	73

**Table 4:** Hit ratios (%) for model 4

Sample size	MAF	Method	Variance				
			0.1	0.2	0.3	0.4	0.5
200	0.2	GFQMDR	76	36	17	6	3
		FQMDR	76	41	21	8	3
		OMDR	69	41	21	10	4
		MDR	65	39	17	3	2
	0.4	GFQMDR	86	65	49	30	17
		FQMDR	83	60	35	17	12
		OMDR	85	56	42	17	9
		MDR	76	50	24	13	6
400	0.2	GFQMDR	88	50	18	7	3
		FQMDR	85	61	33	15	7
		OMDR	69	47	35	19	12
		MDR	80	59	26	13	5
	0.4	GFQMDR	95	78	56	35	24
		FQMDR	95	66	46	28	22
		OMDR	96	73	46	35	25
		MDR	90	57	37	26	16
800	0.2	GFQMDR	98	74	45	22	10
		FQMDR	98	77	46	27	19
		OMDR	88	61	33	26	15
		MDR	95	71	45	25	14
	0.4	GFQMDR	1	90	74	59	48
		FQMDR	1	87	65	45	37
		OMDR	1	91	63	46	34
		MDR	1	74	57	44	36

**Table 5:** Hit ratios (%) for model 5

Sample size	MAF	Method	Variance				
			0.1	0.2	0.3	0.4	0.5
200	0.2	GFQMDR	83	48	29	10	3
		FQMDR	79	38	11	2	3
		OMDR	85	39	15	5	2
		MDR	71	31	12	2	0
	0.4	GFQMDR	82	56	38	25	11
		FQMDR	75	51	32	16	7
		OMDR	76	50	34	17	8
		MDR	72	42	22	8	6
400	0.2	GFQMDR	94	78	53	26	20
		FQMDR	93	59	22	14	6
		OMDR	97	62	33	18	6
		MDR	90	52	25	8	5
	0.4	GFQMDR	94	78	56	36	23
		FQMDR	93	68	46	25	15
		OMDR	96	64	45	34	22
		MDR	90	64	35	16	8
800	0.2	GFQMDR	99	90	73	58	39
		FQMDR	99	75	55	35	22
		OMDR	100	86	50	36	28
		MDR	98	60	47	25	18
	0.4	GFQMDR	1	94	76	59	45
		FQMDR	1	91	66	45	37
		OMDR	1	86	63	50	36
		MDR	1	82	51	47	35

The performance of GFQMDR is better than other three methods in general. It is also observed that the performances of FQMDR and OMDR are better than that of MDR and the performance of FQMDR is slightly better than that of OMDR.

For the type I error rate, results given in Table 6 show that GFQMDR has type I error rate tightly gathering around 1% with a range from 0.7 to 1.3%, better than three other methods. Therefore GFQMDR controls type I error rate better.

**Table 6:** Type I Error Rate with the Significance Level 훼 of 0.01 from Datasets with 1000 Replicates

*m*	Method	*n*		
		200	400	600
10	GFQMDR	1.2%	1.2%	1.2%
	FQMDR	1.2%	0.4%	1.1%
	OMDR	1.8%	0.8%	1.7%
	MDR	1%	0.3%	1.3%
15	GFQMDR	1.2%	0.8%	1.3%
	FQMDR	0.8%	0.5%	0.7%
	OMDR	0.8%	0.6%	0.6%
	MDR	0.8%	0.6%	1.5%
20	GFQMDR	1.1%	1.1%	0.7%
	FQMDR	0.8%	1.2%	1.7%
	OMDR	0.6%	1.4%	1.3%
	MDR	0.9%	1%	1.1%

Tables 7 and 8 show results for the third experiment. All methods identify both models with relatively high ratios when two weights are similar and identify the model having higher weight with high ratios and the model having lower weight with low ratios when two weights are different. On the whole, GFQMDR identifies model 2, 3 and 4 with higher hit ratios than three other methods, but identifies model 1 with lower hit ratios than FQMDR and OMDR.

**Table 7:** Hit ratios (%) for model 1 and model 2

Weight (*w*_1_:*w*_2_)	MAF	Method	Variance		
			0.1	0.2	0.3
0.5:0.5	0.2	GFQMDR	33:40	3:21	1:5
		FQMDR	59:9	15:5	3:2
		OMDR	52:7	13:9	4:4
		MDR	48:8	8:3	2:1
	0.4	GFQMDR	20:85	21:47	18:18
		FQMDR	19:80	25:37	15:19
		OMDR	23:75	26:45	20:22
		MDR	20:71	19:34	10:13
0.7:0.3	0.2	GFQMDR	90:0	46:0	8:0
		FQMDR	87:0	56:0	18:0
		OMDR	83:0	40:0	18:2
		MDR	89:0	43:0	6:0
	0.4	GFQMDR	100:4	96:4	82:0
		FQMDR	100:4	97:3	79:1
		OMDR	100:7	96:6	78:2
		MDR	100:0	91:1	66:1
0.3:0.7	0.2	GFQMDR	0:85	0:50	0:22
		FQMDR	3:53	1:24	0:5
		OMDR	3:47	0:25	0:14
		MDR	1:45	0:14	0:3
	0.4	GFQMDR	0:98	0:80	0:47
		FQMDR	0:97	0:79	0:46
		OMDR	0:90	0:72	0:46
		MDR	0:95	0:73	0:35

**Table 8:** Hit ratios (%) for model 3 and model 4

Weight (*w*_1_:*w*_2_)	MAF	Method	Variance		
			0.1	0.2	0.3
0.5:0.5	0.2	GFQMDR	84:0	35:0	8:0
		FQMDR	70:1	14:0	3:0
		OMDR	67:0	16:2	7:0
		MDR	62:0	6:1	2:0
	0.4	GFQMDR	96:28	75:12	44:4
		FQMDR	94:30	73:11	46:3
		OMDR	92:28	70:9	40:5
		MDR	93:16	65:7	32:1
0.7:0.3	0.2	GFQMDR	94:0	65:0	41:0
		FQMDR	91:0	47:0	12:0
		OMDR	84:0	50:0	22:0
		MDR	90:0	37:0	7:0
	0.4	GFQMDR	100:0	100:1	96:1
		FQMDR	100:0	100:0	96:1
		OMDR	100:0	100:0	92:0
		MDR	100:0	100:0	84:0
0.3:0.7	0.2	GFQMDR	7:51	3:11	1:7
		FQMDR	3:30	1:4	0:2
		OMDR	6:24	2:5	1:2
		MDR	1:19	0:2	0:0
	0.4	GFQMDR	0:85	1:53	2:21
		FQMDR	0:81	1:50	1:17
		OMDR	0:74	0:49	1:17
		MDR	0:69	2:34	2:12

### Experiments on real data

#### Experimental setup

We use two real datasets to show applications and performance of the proposed method.

One is high density lipoprotein and atherosclerosis data of 294 female F2 mice.

Atherosclerosis is a complex disease related to both environmental and genetic factors. Since the QTL for a trait are located in homologous regions in mice and humans, analysis of mouse atherosclerosis can facilitate genetic analysis of human atherosclerosis.

Female B6 mice have low plasma *high-density lipoprotein* (HDL) levels and are susceptible to atherosclerosis while female 129 mice have high plasma HDL levels and are relatively resistant. F2 mice are derived from intercross of (B6 × 129) F1 progeny produced by the mating of C57BL/6 J (B6) and 129S1/SvImJ (129) mice. This dataset contains genotypes of 111 SSLPs, HDL concentration and size of aortic fatty streak measurements for 294 female F2 mice fed a high-fat diet for 14 weeks [37]. The data were downloaded from the Center for Genome Dynamics at the Jackson Laboratory https://phenome.jax.org/projects/Ishimori1. Here HDL concentrations and size of *aortic fatty streak* (AFS) measurements are two quantitative traits of interest. The atherosclerotic aortic fatty streak lesion size variable was logarithmically transformed (base 10).

Another is *Ultra-violet* (UV) Light-Induced Immunosuppression Data. F1 backcross mice are derived from a backcross between low susceptibility BALB/c female mice and high susceptibility (BALB/c × C57BL/6) F1 male mice. This dataset contains 64 markers, sex and UV light-induced *percent immunosuppression* (PI) of a contact hypersensitivity response of 134 F1 backcross mice. The data were acquired from the Center for Genome Dynamics at the Jackson Laboratory https://phenome.jax.org/projects/Clemens1. UV light-induced percent immuno- suppression is the quantitative trait of interest.

For missing values of SSLP, we set them to the majority value of that SSLP; for missing values of QTs, we set them to the mean value of that quantitative trait.

All three QTs are divided into three equal length intervals since better performance can be achieved in this way. For HDL concentrations, three intervals are defined as high concentration, middle concentration and low concentration states respectively; for size of AFS, three intervals are defined as large size, middle size and small size states respectively; for UV light-induced percent immunosuppression three intervals are defined as high percent immunosuppression, middle percent immunosuppression and low percent immunosuppression states respectively.

#### Experiment results

The GFQMDR method is used to select multiple best 2-way, 3-way and 4-way interactions in the above real datasets associated with HDL, AFS and PI respectively and α is set as 0.01 in step 8 of the GFQMDR algorithm here.

The performance of the GFQMDR method is evaluated in maximum testing balanced classification accuracy (MTSBCA) on ten CVs and corresponding CVC, where CVC is used as a tie break, and compared with that of FQMDR, OMDR and MDR methods. Balanced accuracy using the extended linear member function, balanced accuracy using the traditional linear member function, tau-b and balanced accuracy are used to select multiple sets of best interaction SNPs in each CV in GFQMDR, FQMDR, OMDR and MDR methods respectively. We choose multiple best sets of SNPs for each of 2-way, 3-way and 4-way interactions.

We set k to 3, i.e. for each CV of a specific QT, we choose three best sets of SNPs of a fixed order. MTSBCA1 through MTSBCA3 are used to represent three sets of SNPs which have largest MTSBCAs in the descending order and GCVC1 through GCVC3 are corresponding GCVCs which are used as a tie break.

**Fig. 4:**
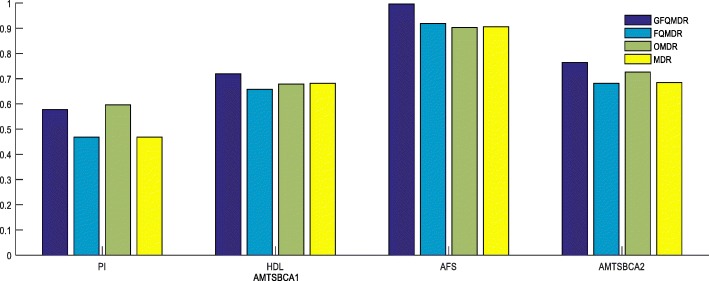
Comparison of AMTSBCA1 (average maximum testing balanced classification accuracy of a trait), AMTSBCA2(average maximum testing balanced classification accuracy of all traits) among GFQOMDR, FQMDR, OMDR and MDR

From Tables 9, 10 and 11, we can see that the performance of GFQMDR is better than that of FQMDR, OMDR and MDR in most cases. Figure 4 shows that AMTSBCA1 with GFQMDR is higher than that with three other methods for each of the four QTs except for PI with OMDR, AMTSBCA2 with EFQMDR is higher than that of three other methods.

**Table 9:** Comparison of MTSBCA and GCVC of PI classifiers among EFQMDR, FQMDR, OMDR and MDR when k = 3

Classifier	Two loci				Three loci				Four loci			
Method	EFQMDR	FQMDR	OMDR	MDR	EFQMDR	FQMDR	OMDR	MDR	EFQMDR	FQMDR	OMDR	MDR
MTSBCA1	0.563	0.597	0.583	0.456	0.657	0.45	0.857	0.514	0.783	0.514	0.651	0.583
MTSBCA2	0.488	0.542	0.5	0.45	0.488	0.4	0.625	0.411	0.783	0.5	0.613	0.5
MTSBCA3	0.4	0.458	0.467	0.413	0.488	0.333	0.478	0.389	0.540	0.422	0.590	0.5
GCVC1	5	8	3	3	5	2	7	2	8	1	1	4
GCVC2	5	6	3	5	2	1	9	3	7	1	1	1
GCVC3	1	1	2	2	1	2	1	1	1	1	4	1

**Table 10:** Comparison of MTSBCA and GCVC of HDL classifiers among EFQMDR, FQMDR, OMDR and MDR when k = 3

Classifier	Two loci				Three loci				Four loci			
Method	EFQMDR	FQMDR	OMDR	MDR	EFQMDR	FQMDR	OMDR	MDR	EFQMDR	FQMDR	OMDR	MDR
MTSBCA1	0.778	0.734	0.778	0.716	0.796	0.667	0.667	0.685	0.648	0.675	0.671	0.833
MTSBCA2	0.778	0.685	0.778	0.716	0.796	0.667	0.667	0.667	0.611	0.593	0.671	0.657
MTSBCA3	0.778	0.685	0.547	0.704	0.759	0.622	0.667	0.541	0.526	0.593	0.668	0.620
GCVC1	10	10	7	10	2	4	3	1	2	4	4	1
GCVC2	8	10	6	9	1	1	3	3	2	1	2	2
GCVC3	4	5	3	5	2	1	2	3	1	1	4	3

**Table 11:** Comparison of MTSBCA and GCVC of AFS classifiers among EFQMDR, FQMDR, OMDR and MDR when k = 3

Classifier	Two loci				Three loci				Four loci			
Method	EFQMDR	FQMDR	OMDR	MDR	EFQMDR	FQMDR	OMDR	MDR	EFQMDR	FQMDR	OMDR	MDR
MTSBCA1	1	0.862	0.828	0.857	1	0.982	0.931	0.939	1	0.970	1	1
MTSBCA2	1	0.839	0.793	0.759	1	0.982	0.911	0939	1	0.970	1	1
MTSBCA3	1	0.821	0.788	0.759	1	0.875	0.911	0.897	0.966	0.966	0.966	1
GCVC1	9	3	5	5	4	7	3	4	3	4	4	4
GCVC2	8	8	3	5	4	4	3	3	3	2	4	4
GCVC3	7	7	4	4	2	2	3	2	2	2	1	2

After computing *P* values of these classifiers, we found that HDL has *P* values 0 for all three classifiers of two way and three way classifiers for GFQMDR and FQMDR, 0, 0.002, 0.002 for three classifiers of two way classifiers for OMDR, 0, 0.001, 0.001 for three classifiers of two way classifiers for MDR, 0.001, 0.003, 0.004 for three classifiers of three way classifiers for OMDR. In other cases, *P* values are all above 0.01. For *P* values below 0.01, GFQMDR and FQMDR have lower *P* values than OMDR and MDR, indicating stronger gene-gene interactions.

For cases where *P* values are below 0.01, we set *k* to bigger values and identify more classifiers with *P* values below 0.01, but some of them are identified due to linkage disequilibrium among causal snps and non-causal snps.

In summary the performance of the proposed algorithm is better than that of FQMDR, OMDR and MDR.

## Discussion

In step 3 and step 4 of GFQMDR Algorithm, when computing the size of each category in a particular cell, an extended linear member function is used; when computing the total size of each category in all cells, a traditional linear member function is used. The reason is that when deciding the label or category of a particular cell, the difference among different categories when being tried to assign to that cell can be reflected by the size of different categories in that cell, rather than the total size of different categories in all cells. Such a difference can be better reflected by an extended linear member function. Experiments also show much better performance when using the extended linear member function and the traditional linear member function in different cases.

In GFQMDR Algorithm, fuzzification is not only applied to the computation of training and testing accuracies, but also applied to the classification of each cell or genotype combination, while in [34], fuzzification is only applied to the computation of training and testing accuracies.. Experiments show better performance of such a double fuzzification than that of a single fuzzification in either the computation of training and testing accuracies or the classification of each cell or genotype combination.

It’s a complex problem to divide QTs into meaningful intervals. Usually deviation is used to divide QTs as in simulated data, but the condition is the data obey approximately some kind of normal distribution. If not, dividing QTs into equal length intervals is a simple and acceptable choice if it has a better performance.

To make our method more computationally efficient, the GENIE software package which utilizes the power of multiple GPU or CPU processor cores to parallelize the interaction analysis [38] could be used.

Alternative methods could be to use fuzzy balanced accuracy based on traditional member function of fuzzy sets, or balanced signed accuracy where 1 is used to denote that the predicted category is the same as the true category, 0 to denote that the predicted category is close to the true category, − 1 to denote that the predicted category is far from the true category. However our experiments show that the performance of our algorithm is better than that of the above two methods.

When multiple sets of causal snps exist, the performance of our proposed method depends on the sizes of influence of different sets of causal snps. When the sizes are similar, they are easier to be identified, whereas the sizes are quite different, the set with bigger size will be easily identified.

Mathematical analysis is further needed to explain the better performance of the generalized fuzzy classification based on extended member functions. This will be our future work.

To apply our method, the continuous trait should be divided into J intervals first. To get the optimal J, we can try different number of intervals. If for J intervals, its performance is better than J-1 intervals and J + 1 intervals, J intervals could be approximately considered as optimal. If the performance is increasingly better when J increases, we can set an upper bound. In this paper, we only try three intervals for simplicity. We intend to try more intervals in our future work.

We would also try testing the proposed method with data in dbGAP or other human data that we can get a hold on in our future work.

## Conclusions

In this study, a new method to identify gene-gene interactions for complex quantitative traits is proposed based on generalized fuzzy classification. To better use the information contained in a quantitative trait, it is first divided into several (greater than two) ordinal levels. Then a new ordinal association measure, fuzzy balanced accuracy based on generalized fuzzy classification is employed to select best sets of SNPs as having the strongest association with the trait in our proposed GFQMDR algorithm. Experimental results on simulated datasets and real datasets show that our algorithm has better performance in identifying gene-gene interactions associated with a complex quantitative trait.

## Abbreviations

AFS: Aortic fatty streak
CV: Cross validation
FQMDR: Fuzzy quantitative MDR
GFQMDR: Generalized fuzzy quantitative MDR
GWAS: Genome-wide association study
HDL: High-density lipoprotein
LD: Linkage disequilibrium
MAF: Minor allele frequencies
MDR: Multifactor dimensionality reduction OMDR Ordinal MDR
PI: Percent immunosuppression
QT: Quantitative trait
SNP: Single nucleotide polymorphism
